# Effects of Virtual Reality-Based Therapy on Quality of Life of Patients with Subacute Stroke: A Three-Month Follow-Up Randomized Controlled Trial

**DOI:** 10.3390/ijerph18062810

**Published:** 2021-03-10

**Authors:** Marta Rodríguez-Hernández, Juan-José Criado-Álvarez, Ana-Isabel Corregidor-Sánchez, José L. Martín-Conty, Alicia Mohedano-Moriano, Begoña Polonio-López

**Affiliations:** 1 Faculty of Health Sciences, University of Castilla La Mancha, 45600 Talavera de la Reina, Spain; Marta.RHernandez@uclm.es (M.R.-H.); AnaIsabel.Corregidor@uclm.es (A.-I.C.-S.); JoseLuis.MartinConty@uclm.es (J.L.M.-C.); Alicia.Mohedano@uclm.es (A.M.-M.); Begona.Polonio@uclm.es (B.P.-L.); 2 Technological Innovation Applied to Health Research Group (ITAS), Faculty of Health Sciences, University of Castilla La Mancha, 45600 Talavera de la Reina, Spain; 3 Department of Public Health, Institute of Health Sciences, 45600 Talavera de la Reina, Spain

**Keywords:** stroke, virtual reality, quality of life, occupational therapy, randomized controlled trial

## Abstract

Objective: To evaluate the influence of conventional rehabilitation combined with virtual reality on improving quality of life related to post-stroke health. Design: Randomized controlled trial. Setting: Rehabilitation and neurology departments of a general hospital (Talavera de la Reina, Spain). Subjects: A total of 43 participants with subacute stroke. Intervention: Participants were randomized into experimental group (conventional treatment + virtual reality) and control (conventional treatment). Main measures: Health-related quality of life as measured by the EuroQoL-5 dimensions instrument (EQ-5D-5L) and EuroQoL visual analog scale (EQ-VAS). Results: A total of 23 patients in the experimental group (62.6 ± 13.5 years) and 20 in the control (63.6 ± 12.2 years) completed the study. In the experimental group, EQ-VAS score was 29.1 ± 12.8 at baseline, 86.5 ± 7.1 post-intervention, and 78.3 ± 10.3 at the three-month follow-up. The control group obtained scores of 25.5 ± 5.1, 57.0 ± 4.7, and 58.5 ± 5.9, respectively. We identified significant differences at the post-intervention and follow-up timepoints (*p* = 0.000) and a partial η^2^ of 0.647. In EQ-5D-5L, the severity of issues decreased after intervention in the experimental group, while pain and anxiety dimensions increased between post-intervention and follow-up. Conclusions: The conventional rehabilitative approach combined with virtual reality appears to be more effective for improving the perceived health-related quality of life in stroke survivors.

## 1. Introduction

Stroke is one of the world’s leading health problems and a major cause of acquired disability in adults [1,2]. Motor and sensory deficits are common in stroke patients, producing disorders of motor control, balance, and gait [3]. An estimated 80% of the survivors present motor difficulties in the upper extremities, affecting their execution of activities of daily living, performance in the community, and health-related quality of life (HRQoL) [4,5,6]. In addition, alterations in body alignment occur, requiring the incorporation of treatment strategies focused on improving the postural control and symmetry of weight bearing [7,8,9]. The multidimensional impact of the diagnosis on survivors is of vital importance for their functional recovery, and cannot be explained exclusively by clinical variables. Measures of perceived health status, social participation, and HRQoL are increasingly used as part of a person-centered assessment process, reflecting the interaction between health determinants and physical, social, and emotional functions [10,11]. HRQoL is an important factor, together with the increase in life expectancy, the chronification of pathologies, and the achievement of health policies focused on the patient’s subjective wellbeing and satisfaction after completing the intervention processes [2,12,13,14]. The EuroQoL-5 dimensions instrument (EQ-5D-5L) is useful for evaluating HRQoL over time, comparing the evolution between groups and calculating the cost-effectiveness and/or effectiveness of the administered interventions [15,16].

In recent years, the use of neurorehabilitation approaches based on technology and virtual reality (VR) has increased, allowing the creation of effective rehabilitation environments and providing multimodal, controllable, and customizable stimulation [17]. In these simulations, the recreation of virtual objects maximizes visual feedback [18], and a high intensity and high number of repetitions fundamentally influence neuroplasticity and functional improvement of the patients [19]. VR-based rehabilitation offers the possibility to individualize treatment needs and, at the same time, standardize assessment and training protocols [20,21]. In this sense, it should be noted that virtual reality technology, specifically for rehabilitation processes for people with neurological pathologies, makes it possible to work in a functional way and with specific intervention objectives, as well as to easily qualify and document the progress made during the session [22].

The present study aims to examine the effect of a combined treatment of conventional therapy with virtual reality on HRQoL of people diagnosed with stroke in the acute phase and its evolution after three months in the integrated health area of Talavera de la Reina.

## 2. Materials and Methods

### 2.1. Study Design

This study began in April 2018 and was completed by March 2020. It adhered to the standards of the Declaration of Helsinki and was approved by the Research Ethics and Medicines Committee (CEIm) of the integrated health area of Talavera de la Reina (12/2018). The study was registered in the trial registry ISRCTN (ISRCTN27760662) [23]. All participants received verbal and written information about the study and gave their written informed consent.

This randomized controlled trial compared conventional rehabilitation of physical therapy and occupational therapy (control group) and the combination of conventional rehabilitation with the use of virtual reality technology (experimental group), following the guidelines of the Consolidated Standards of Reporting Trials (CONSORT) [24]. The change in HRQoL (baseline, post-intervention, and three-month follow-up) was used as the primary outcome. The evaluation of the post-intervention variables was completed three weeks after the start of treatment in both groups (after receiving 15 treatment sessions).

The participants were recruited from the neurology and rehabilitation units of the University General Hospital of Talavera de la Reina, Spain. They were assigned randomly to the control or experimental group by an external researcher to the intervention and evaluation process (allocation ratio of 1:1). The conventional rehabilitation therapists were blinded to the study, but the intervention could not be blinded to the participants or to the therapist who applied the combined treatment.

### 2.2. Participant and Settings

The study included 46 patients (of whom 43 completed the intervention period and follow-up evaluation) who attended the hospital rehabilitation unit (mean age: 63.1; % of women: 18.6), who had been diagnosed with stroke and met the following inclusion criteria: (1) age: 18 to 85 years; (2) maximum evolution time of six months; (3) upper limb motor involvement; (4) dependence in activities of daily living (Stroke Impact Scale; version 3.0); (5) life expectancy greater than six months (absence of life-threatening diagnoses such as end-stage cancer); and (6) absence of other serious and disabling pathologies. Four exclusion criteria were defined: presence of other neurological diagnoses, severe hemineglect, psychiatric pathologies, and signed revocation of informed consent.

### 2.3. Intervention

All study participants received 15 treatment sessions with a duration of 150 min per session over five consecutive days per week. In total, the intervention lasted three weeks per participant. The patients assigned to the experimental group combined conventional upper and lower limb strength and motor training (100 min; guided by the hospital’s physiotherapy and occupational therapy team) with the use of virtual reality technological devices (50 min), while the participants in the control group received conventional physiotherapy and occupational therapy of 75 min each.

The conventional intervention protocol consisted of receiving manual therapy techniques (massage); passive and active assisted mobilization of the upper and lower limbs; walking in parallel, on slope and stairs; exercises with and without resistance with balls, elastic bands, and dumbbells in therapeutic cage and trellises; active-assisted mobility exercises of the upper limb and fingers in a sitting position; moving objects horizontally on a table; elevation and superposition of objects in vertical plane; biomechanical tasks that simulate flexion–extension and abduction–adduction of the shoulder and flexion–extension of the wrist and fingers.

**Figure 1:**
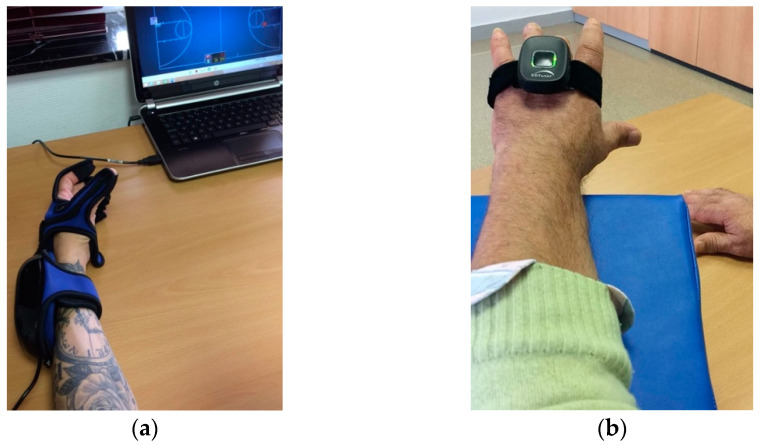
(**a**) HandTutor© glove (flexion–extension of the fingers); (**b**) 3DTutor© (wrist extension).

Motor training with virtual reality devices consisted of several systems: First, the HandTutor© glove (Figure 1a) [25,26] for hand rehabilitation and 3DTutor© (Figure 1b) for upper extremities [21]. Both systems are based on intensive and repetitive practice through movement and feedback instructions provided by the software with virtual environments and tasks that simulate movements that stroke survivors require for daily life [27,28,29]. The glove allows the blocking of compensatory joint movements and ensures a better adaptation to functional tasks (for example, active joint range movement can be limited). It focuses on flexion–extension of fingers and wrist [30]. The 3DTutor© allows the therapist to focus on training all movements of the head, trunk, shoulder, elbow, wrist, hip, knee, and ankle joints [31]. Second, the Rehametrics© software [32] + Microsoft Kinect sensor [33,34,35] was used for the recovery of the upper limb (elbow and shoulder), trunk, and lower body, which simulates activities of daily living and mobility in the community with virtual environments and in combination with the use of gamification. It monitors and captures the user’s movement in real time and allows the therapist to adjust and customize the difficulty, duration, range of motion, and the number of distracting or visual aids.

The baseline and post-interventional assessments and the three-month follow-up were conducted by the same researcher in both groups.

### 2.4. Outcomes Measures

The primary outcome variable for this study was HRQoL. For its quantification, we applied the EQ-5D-5L instrument, which is widely used in the literature [36]. It is quick and easily applied, validated in Spanish, and reliable in both healthy and symptomatic populations [37,38]. It consists of five dimensions (descriptive system) that assess mobility, personal care, activities of daily living, pain/discomfort, and anxiety/depression, assigning to each dimension the following response categories (level of severity): no problems, mild/moderate problems, and severe problems/inability to perform the activity. In addition, it uses the EuroQoL visual analog scale (EQ-VAS) for self-assessment of the state of improvement and/or health condition, in which it scores from 0 (minimum) to 100 (maximum). The reliability of the EQ-5D-5L test was r = 0.90 and that of EQ-VAS r = 0.86 [39,40]. In addition, sociodemographic and clinical data were recorded, such as age, sex, time elapsed since diagnosis, location of the lesion, risk factors, dominance, pain, or mild hemispatial neglect syndrome. HRQoL was recorded before starting the treatment (baseline), three weeks after the start (post-intervention), and three months after its completion (follow-up).

### 2.5. Statistical Analysis

The sample size was calculated using the Epidat 4.2 program. An effectiveness ratio of 90% was estimated for the experimental group and 50% for the control group. Using a power of 80% and a confidence level of 95% (*p* < 0.05), a minimum sample size of 20 participants was obtained in each group. The data were analyzed with the IBM SPSS statistical package (version 24.0) (IBM, S.A., Madrid, Spain.). To compare the clinical and sociodemographic variables of the intervention groups, Student’s t and chi-square tests were used. Differences in baseline HRQoL, post-intervention, and three-month follow-up scores were analyzed with inter- and intragroup ANOVA, Student’s t test, and the chi-square test. The clinically relevant improvement was defined by Robinson et al. [41] as 0.05 points [42]. Statistical significance was set at a *p*-value below 0.05.

The analysis of missing data from the control group was carried out with multiple imputation in the analysis (expectation maximization and regression method), with a little’s chi-square statistic 14,247 (degree freedom = 19; *p* = 0.769).

The investigator who performed the statistical analysis was unaware of the random assignment of participants to the intervention groups.

## 3. Results

**Figure 2:**
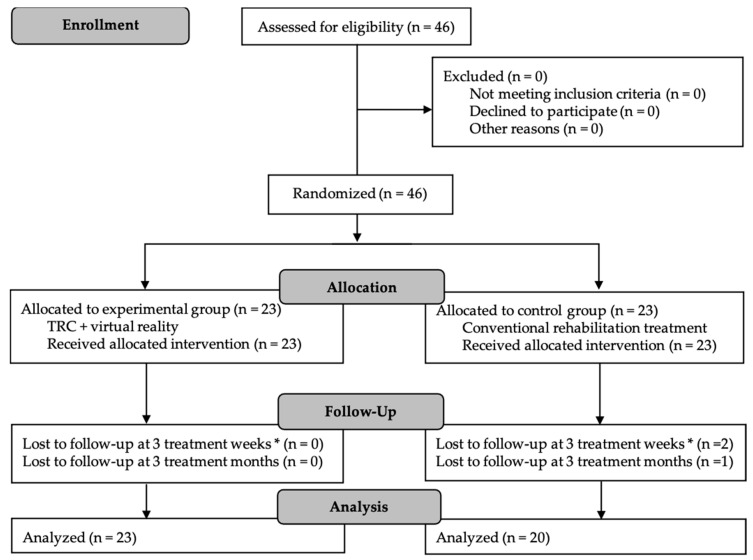
CONSORT Flow Diagram for participant recruitment, allocation, follow-up and analysis. TRC: conventional rehabilitation treatment. * Post-intervention evaluation.

A total of 46 patients were selected for randomization, of whom 43 completed the intervention period and follow-up evaluation. A total of 23 participants were assigned to the experimental group and 23 to the control group. Three participants were lost in the control group, as a consequence of the start of the COVID-19 pandemic in Spain (Figure 2).

The sociodemographic and clinical characteristics of the participants are shown in Table 1. Significant differences are observed in the evolution of pain between both groups, which decreased considerably after the intervention in the experimental group. A total of 15.0% (n = 3) of the participants in the control group registered a change in dominance (from right to left) post-intervention, while the experimental group maintained baseline dominance. A total of 43.5% (n = 10) in the experimental group and 30.0% (n = 6) in the control group were diagnosed with arterial hypertension, which thus constituted the main risk factor.

**Figure 3:**
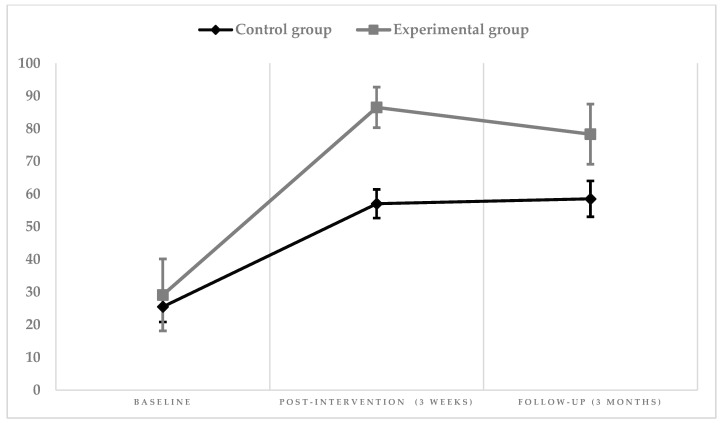
Graphic representation of the intervention’s effect on EQ-VAS.

Table 2 shows the differences in the evolution of EQ-VAS results between groups. An increase of the score is observed in both groups; however, it was notably higher in the experimental group (mean score at baseline 29.1 to post-intervention 86.5). During follow-up, the mean score of the control group remained stable, whereas the score in the experimental group decreased slightly (post-intervention 86.5 to follow-up 78.3) (Figure 3).

The magnitude of the effect of the experimental intervention is large and statistically significant.

The differences in the EQ-5D-5L HRQoL results between groups are shown in Table 3. We observed an effect of the experimental intervention on the frequency distribution in all dimensions, with statistically significant differences between baseline, post-intervention, and follow-up, except for pain/discomfort (baseline: 0.562; three-month follow-up: 0.147). Marked was the increased severity of issues of pain and anxiety dimensions of the experimental group between the post-intervention and the follow-up (pain: from 21.7 to 82.6; anxiety: from 8.7 to 65.2).

## 4. Discussion

This randomized controlled trial examined the effects of treatment based on virtual reality combined with conventional rehabilitation for stroke patients. An objective and validated outcome measure was used for the assessment of HQRoL [43], compared to a control group. Our results show that the use of a virtual reality program combined with conventional therapy produces significant changes in HQRoL in stroke survivors in the subacute phase. However, the results of the present study cannot be extrapolated to stroke patients in other stages of the disease or to rehabilitation systems with less-intensive intervention programs or in which the start of treatment is delayed.

Regarding the HQRoL, our results indicate that the combination of conventional treatment with a virtual semi-immersive approach produces positive effects in reducing the severity of problems in the dimensions of the EQ-5D-5L and in improving subjectively perceived health status (EQ-VAS). We highlight the effectiveness of the experimental intervention in the perception of pain and in the sensation of anxiety or depression, coinciding with another study [44]. A recent meta-analysis correlated post-stroke depression with a significantly higher risk of mortality in stroke patients [9].

The significant improvements of the participants in the experimental group can be explained theoretically. Task-oriented repetitive practice may provide more effective motor relearning for post-stroke neuronal recovery [45]. In this sense, the virtual reality devices used for this randomized controlled trial focus on intensive hand and upper limb movements, forcing the patient to mobilize body segments in various directions and at different speeds. The virtual reality games simulate movements necessary for the execution of activities of daily life. The patients in the experimental group experienced changes in their lives before and after the virtual reality session. Intensive and repetitive mobilization increased the activation of the affected area, reducing disuse and allowing the patients to carry out tasks outside the hospital setting with greater independence. The improved execution of movement and the increased functional independence may explain the decrease or absence of pain in the most affected upper limb and the results obtained in the anxiety–depression dimension of the EQ-5D-5L instrument.

It would therefore seem beneficial to establish lines of research and treatment programs aimed at reducing anxiety and pain in these patients. Along these lines, the use of virtual reality can be an interesting tool to include in protocols adapted to stroke patients [21]. The differences observed in the subjective diminution of problems performing activities of daily living stand in contrast to the minimum number of sessions established in other studies for this type of intervention [46,47]; however, they coincide with experimental interventions based on the adjustment of the balance between challenge and skill [42], an implicit characteristic in the specific virtual reality devices used for randomized controlled trials. In this sense, it should be noted that most of the published studies use commercial games or video consoles [48].

Current findings indicate that structural changes occur in response to motor learning during adulthood. Rehabilitative approaches that involve a high intensity of practice, positive feedback between stimulus and response, and increased motivation [29] are associated with more positive changes, without implying more hours of training [49,50]. Understanding the mechanisms of brain plasticity after injury is key to optimizing survivor interventions, promoting adaptive structural plasticity from the outset [30,51,52,53]. Based on our results, we can affirm that the use of virtual reality can be an appropriate complement for the conventional rehabilitation of patients with stroke in the subacute phase. Preventing and reducing pain in the most affected body segments, increasing the feeling of wellbeing, and improving the perception of the quality of life of the people with whom we work can allow an optimal recovery of the stroke survivor and his family, having repercussions, in turn, in the reduction of health and community services after the end of the subacute phase.

This research supports the integration of virtual reality in clinical practice and in the improvement of HQRoL of stroke survivors in the subacute phase. These systems can be incorporated into conventional intervention programs in stroke patients as adjunctive treatment [54,55].

Limitations of this study were that it did not include a long-term follow-up to determine if the differences within and between groups are maintained after the completion of the subacute phase. This long-term follow-up has not been possible due to the start of the COVID-19 pandemic in Spain. The present study was limited to a single center which could increase the effect of treatment, compared to other multicenter randomized controlled trial designs. The participants could not be blinded because they coincided in the conventional rehabilitative treatment and transmitted the evolution and improvements due to the use of virtual reality systems in hand mobility, reduction of shoulder pain, and improvement in the performance of activities of daily living [56,57].

## 5. Conclusions

The conventional rehabilitative approach combined with virtual reality would appear to be more effective than conventional treatment alone for improving the evaluated and perceived health-related quality of life in stroke survivors. We highlight two clinical messages: (1) Virtual reality as complement to conventional rehabilitation treatment is associated with a perceived increase of HRQoL in stroke survivors, and (2) three months after finishing the treatment, the effect of the combined intervention is reduced, especially in the dimensions of pain, anxiety, and depression.

## Figures and Tables

**Table 1:** Characteristics of the participants of both groups (n = 43).

Study Variables	Experimental Group (n = 23)	Control Group (n = 20)	Difference of Mean between Groups (*p*-Value)
Age			
Mean (SD) Below 55 years (%) 55 to 70 years (%) Above 70 years (%)	62.6 (13.5) 26.1 30.4 43.5	63.6 (12.2) 25.0 45.0 30.0	−0.9 (*0.812*) *0.566*
Sex			
Male Female	78.3 21.7	85.0 15.0	*0.571*
Main diagnostic			
Ischemic/thrombotic Hemorrhagic	91.3 8.7	90.0 10.0	*0.883*
Middle cerebral artery lesion (%)	60.9	55.0	*0.697*
Time since diagnostic (days) *			
Baseline (pre-intervention) Post-intervention (3 week follow-up) Follow-up (3 months)	55.3 (34.3) 75.3 (34.3) 162.3 (36.9)	54.2 (30.4) 74.2 (30.4) 157.2 (36.1)	1.1 (*0.909*) 1.1 (*0.909*) 5.1 (*0.650*)
Hemispatial neglect syndrome (%)	13.0	10.0	*0.756*
Presence of pain in extremities			
Baseline (pre-intervention) Post-intervention (3 week follow-up) Follow-up (3 months)	43.5 21.7 82.6	50.0 80.0 100.0	*0.669* *0.000* *0.050*
Location of the brain injury (%)			
Right Left	82.6 17.4	85.0 15.0	*0.832*
Dominance (change baseline to post-intervention)			
Right Left	100.0/100.0 0	100.0/85.0 0/15.0	*0.054*

* Mean (SD). *p*-value: Student’s t-test for independent samples in continuous variables/Pearson’s chi-squared test.

**Table 2:** Linear model. Effect of the intervention on EuroQoL visual analog scale (EQ-VAS) results.

Intervention Group				Difference Follow-Up/Baseline			
	Baseline Mean (SD)	Post-Intervention Mean (SD)	Follow-Up Mean (SD)	Mean (CI95%)	ANOVA		
					F	*p*	η^2^ Parcial
EQ-VAS (T-score)							
Experimental group	29.1 (12.8)	86.5 (7.1)	78.3 (10.7)	49.2 (42.2–56.0) *			
Control group	25.5 (5.1)	57.0 (4.7)	58.5 (5.9)	33.0 (29.1–36.8) *	75.2	*0.000*	0.647
*p*	*0.241*	*0.000*	*0.000*				

SD: Standard deviation. CI95%: 95% confidence interval. Partial η^2^: magnitude of effect. * The difference in means is significant at level 0.000. *p*-value: italics.

**Table 3:** Difference in the EuroQoL-5 dimensions instrument (EQ-5D-5L) frequency distribution according to intervention group.

EuroQoL-5 Dimensions Instrument	Baseline (n = 43) %			Post-Intervention (n = 43) %			Follow-Up (n = 43) %		
	CG (n)	EG (n)	*p*	CG (n)	EG (n)	*p*	CG (n)	EG (n)	*p*
D1. Mobility									
(1) (2) (3)	0 60.0 (12) 40.0 (8)	0 21.7 (5) 78.3 (18)	*0.011*	0 90.0 (18) 10.0 (2)	43.5 (10) 56.5 (13) 0	*0.002*	0 90.0 (18) 10.0 (2)	39.1 (9) 60.9 (14) 0	*0.007*
D2. Selfcare									
(1) (2) (3)	0 60.0 (12) 40.0 (8)	0 21.7 (5) 78.3 (18)	*0.011*	20.0 (4) 80.0 (16) 0	60.9 (14) 39.1 (9) 0	*0.014*	25.0 (5) 75.0 (15) 0	65.2 (15) 34.8 (8) 0	*0.014*
D3. Daily activities									
(1) (2) (3)	0 20.0 (4) 80.0 (16)	0 21.7 (5) 78.3 (18)	*0.019*	0 100.0 (20) 0	34.8 (8) 65.2 (15) 0	*0.000*	0 100.0 (20) 0	34.8 (8) 65.2 (15) 0	*0.000*
D4. Pain/discomfort									
(1) (2) (3)	50.0 (10)45.0 (9)5.0 (1)	56.5 (13)26.2 (6)17.3 (4)	*0.562*	0100.0 (20)0	78.3 (18) 21.7 (5) 0	*0.000*	0 95.0 (19) 5.0 (1)	17.4 (4) 82.6 (19) 0	*0.147*
D5. Anxiety/depression									
(1) (2) (3)	0 85.0 (17) 15.0 (3)	0 78.3 (18) 21.7 (5)	*0.090*	0 100.0 (20) 0	91.3 (21) 8.7 (2) 0	*0.000*	0 65.0 (13) 35.0 (7)	30.4 (7) 65.2 (15) 4.4 (1)	*0.005*

CG: control group; EG: experimental group. (1) No problems; (2) mild and moderate problems; (3) severe, extreme problems and incapacity (15). EQ-5D-5L: descriptive system (5 dimensions). *p*-value: chi-squared test.
